# Combined Cataract and Vitrectomy Surgery in Pediatric Patients

**DOI:** 10.3390/medicina61071176

**Published:** 2025-06-29

**Authors:** Armando J. Ruiz-Justiz, Vanessa Cruz-Villegas, Stephen G. Schwartz, Victor M. Villegas, Timothy G. Murray

**Affiliations:** 1 Department of Ophthalmology, University of Puerto Rico School of Medicine, San Juan, PR 00936, USA; vcruz02@msn.com; 2 Department of Ophthalmology, Bascom Palmer Eye Institute, University of Miami Miller School of Medicine, Miami, FL 33136, USA; sschwartz2@med.miami.edu; 3 Miami Ocular Oncology & Retina (MOOR), Miami, FL 33143, USA

**Keywords:** lensectomy-vitrectomy, congenital cataracts, ectopia lentis, retinopathy of prematurity, retinal detachment, persistent fetal vasculature

## Abstract

*Purpose:* To review the current literature on the combined use of cataract surgery (or lensectomy) and vitrectomy in pediatric patients, with a focus on clinical indications, surgical techniques, outcomes, and complications across various pediatric ocular pathologies. *Methods:* A narrative review of published studies addressing the use of combined lensectomy and vitrectomy (LV) in pediatric patients was conducted. Conditions discussed include congenital cataracts, ectopia lentis, retinopathy of prematurity (ROP), retinal detachment (RD), and persistent fetal vasculature (PFV). Key surgical considerations, visual and anatomical outcomes, and postoperative complications were examined. *Results:* The literature search yielded a total of 160 articles, of which 43 met the inclusion criteria and were included in this review. Although lens-sparing vitrectomy (LSV) is preferred in many pediatric cases to preserve accommodation and reduce complications, combined LV is often necessary in advanced or complex diseases. Studies have shown that combined LV can achieve favorable anatomical outcomes, but functional visual recovery remains variable and is affected by factors such as patient age, baseline ocular anatomy, and disease severity. Postoperative complications such as glaucoma, visual axis opacification (VAO), and intraocular lens (IOL) dislocation are more frequent with combined procedures and require long-term follow-up and rehabilitation. *Conclusions:* Combined cataract surgery (or lensectomy) and vitrectomy may represent a valuable strategy in the management of complex pediatric ocular conditions, particularly when individualized to the clinical context. Tailored surgical approaches are essential to optimize anatomic and functional outcomes. Further prospective studies and harmonized multicenter registries are needed to develop evidence-based principles that can guide individualized surgical decision-making in this unique patient population.

## 1. Introduction

The use of combined cataract surgery (or lensectomy) and vitrectomy in pediatric patients is not well-documented in the current medical literature. Although numerous studies have evaluated pars plana vitrectomy (PPV) techniques and outcomes in children, few have specifically addressed the combined approach of cataract extraction or lensectomy with vitrectomy in this population.

Cataract surgery has advanced considerably over the past few decades, particularly with the widespread adoption of phacoemulsification in adult patients. However, pediatric cataract surgery poses unique anatomical and physiological challenges that require different surgical strategies [1]. Unlike adults, phacoemulsification is not the preferred technique for pediatric patients due to the softness of the crystalline lens and the increased risk of complications [1]. In this group, the lens can typically be removed using manual irrigation and aspiration or vitrector-assisted lensectomy, thereby avoiding the need for ultrasonic energy [2]. Moreover, pediatric eyes present with a smaller anterior segment, increased zonular laxity, and a more fragile lens capsule, all of which increase surgical complexity [2]. Additional intraoperative considerations such as primary posterior capsulectomy and anterior vitrectomy are often necessary to reduce the high risk of posterior capsule opacification (PCO) [3].

**Figure 1:**
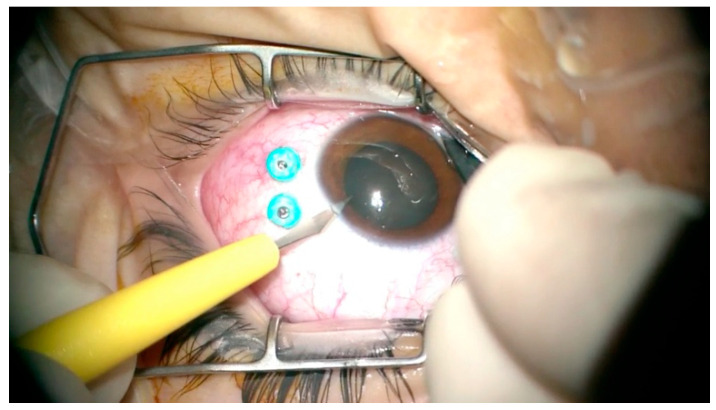
Clear corneal incision in a pediatric patient undergoing combined cataract and vitrectomy surgery.

In complex pediatric ocular conditions, combining cataract extraction or lensectomy (either with or without intraocular lens (IOL) implantation) with vitrectomy is commonly employed, as it may offer significant clinical advantages (Figure 1) [4]. Reported benefits include enhanced visualization of the posterior segment, reduced postoperative vitreous traction, lower rates of macular edema, and the ability to consolidate procedures, thereby decreasing the need for multiple surgical interventions [4]. However, this combined approach carries its own risks, including posterior capsular rupture, zonular dialysis, and posterior dislocation of lens fragments [5].

Several pediatric conditions may necessitate this combined surgical approach, particularly in advanced stages [4]. These include retinopathy of prematurity (ROP), ectopia lentis, retinal detachment (RD), and persistent fetal vasculature (PFV). In such cases, PPV alone or in combination with lensectomy may be required to manage tractional or obstructive pathology [4]. One of the most significant postoperative complications of PPV in pediatric patients is cataract formation, with reported rates as high as 61% [6,7]. This has prompted debate regarding whether a combined lensectomy with IOL implantation and vitrectomy should be preferred over lens-sparing vitrectomy (LSV), particularly in patients at high risk for subsequent cataract development [6,7].

This review aims to summarize the existing literature on the use of combined cataract surgery (or lensectomy) and vitrectomy in pediatric patients, highlighting its indications, advantages, limitations, and clinical outcomes across various pediatric ocular pathologies.

## 2. Materials and Methods

A literature search was performed using the PubMed (https://pubmed.ncbi.nlm.nih.gov/) and Google Scholar (https://scholar.google.com) databases for articles published up to May 2025. Search terms included combinations of: “pediatric cataract”, “lensectomy”, “vitrectomy”, “pars plana vitrectomy”, “lens-sparing vitrectomy”, “lensectomy-vitrectomy”, “pediatric”, “retinopathy of prematurity”, “persistent fetal vasculature”, “ectopia lentis”, and “retinal detachment”.

Prospective and retrospective clinical studies, case series, and relevant review articles were considered. Inclusion criteria were studies published in English that addressed combined lensectomy and vitrectomy (combined LV) techniques in pediatric patients for the management of pediatric cataracts, ectopia lentis, ROP, RD, and PFV. Exclusion criteria included studies focused solely on adult patients, animal models, or those not evaluating lensectomy and vitrectomy procedures.

Due to the heterogeneity of study designs, surgical techniques, and outcome measures across the included literature, a systematic review or meta-analysis was not feasible. Instead, findings were qualitatively summarized to identify trends in surgical decision-making, procedural approaches, and clinical outcomes. Comparative tables were constructed to summarize surgical indications for combined LV, IOL strategies, outcomes, and associated complications.

## 3. Results

The initial literature search yielded a total of 160 articles. After screening titles and abstracts and applying the inclusion and exclusion criteria, 43 articles were deemed relevant and included in this narrative review. Among these, there were 23 retrospective studies, 10 narrative reviews, 4 prospective studies, 3 case reports, 2 systematic reviews, and 1 case series. No randomized controlled trials were identified.

Most studies focused on surgical techniques, visual and anatomical outcomes, and postoperative complications. The majority of clinical studies were conducted at single institutions, with varying sample sizes and follow-up durations.

## 4. Discussion

### 4.1. Pediatric Cataract

Pediatric cataracts remain a leading cause of treatable visual impairment in children worldwide [8]. Prevalence estimates range from 0.63 to 13.6 per 10,000 in low-income countries and 0.42 to 2.05 per 10,000 in high-income countries [3]. They are broadly classified into congenital and acquired types [3]. Early surgical intervention is essential to prevent deprivation amblyopia [9]. Despite consensus on the need for early treatment, the optimal timing and surgical approach for pediatric cataract management continue to be debated.

The importance of technique selection and age-specific surgical planning is highlighted in a retrospective study by Li et al. (2023), which evaluated long-term visual outcomes and complications following lensectomy with anterior vitrectomy and primary IOL implantation in children with bilateral congenital cataracts [10]. The study analyzed 148 eyes from 74 patients who underwent surgery via a limbal approach using a 25-gauge micro-incision vitrectomy system. Surgical steps included lensectomy, anterior vitrectomy, and in-the-bag IOL implantation under general anesthesia. The most common postoperative complications requiring reoperation included visual axis opacification (VAO) (5.4%), IOL pupillary capture (2.0%), iris incarceration (0.7%), and glaucoma (0.7%) [10]. Children younger than 2 years demonstrated a higher incidence of VAO and greater postoperative refractive error compared to older age groups [10]. The mean final best-corrected visual acuity (BCVA) was 0.24 ± 0.32 logMAR, with 22 eyes (14.9%) classified as having low vision (BCVA worse than 0.5 logMAR). These findings suggest that lensectomy with anterior vitrectomy and IOL implantation may be effective and reasonably safe in selected pediatric cases [10]. However, they also underscore the importance of age-specific risk stratification and the need for long-term follow-up, particularly in children under 2 years of age and those with dense cataracts or preexisting comorbidities [10].

VAO is the preferred term over posterior capsule opacification (PCO) in pediatric patients, as visual obscuration can occur despite the creation of a primary posterior capsulorhexis [11]. The underlying pathophysiology of VAO is believed to be either proliferative and/or fibrotic in nature [11]. This includes excessive mitotic activity of residual equatorial lens epithelial cells that migrate into the visual axis, or epithelial-mesenchymal transdifferentiation leading to fibrotic membrane formation. Both mechanisms contribute to visual axis obscuration [11].

Anterior vitrectomy in pediatric cataract surgery plays a pivotal role in reducing postoperative complications, particularly VAO [12]. Kugelberg et al. (2002) demonstrated a statistically significant reduction in reoperation rates due to VAO in children under 7 years old when anterior vitrectomy was performed at the time of cataract surgery [12]. Conversely, a more recent analysis by Yen et al. (2023) did not find age to significantly influence the effectiveness of anterior vitrectomy in preventing VAO, suggesting that the procedure may be beneficial across all pediatric age groups [13].

Postoperative glaucoma is another major complication following cataract surgery in infancy and remains a leading cause of long-term vision loss in this population [14]. Yen et al. (2023) reported that the risk of glaucoma development after anterior vitrectomy and IOL implantation was not significantly associated with patient age [13]. This finding underscores the importance of long-term monitoring for glaucoma in all pediatric patients undergoing cataract surgery, regardless of age or surgical approach [13].

Surgical decision-making in pediatric cataract cases should consider patient age, lens density, and the presence of posterior segment pathology [3,10,11,12,13,14,15]. Lensectomy with anterior vitrectomy is typically recommended in younger children due to the higher risk of VAO [15].

### 4.2. Ectopia Lentis

Ectopia lentis in pediatric patients can result from trauma or may occur secondary to systemic conditions such as Marfan syndrome and other connective tissue disorders [16]. Surgical management of ectopia lentis is complex, and the choice of surgical technique is highly case-dependent [16]. Typically, combined LV is performed, with or without IOL implantation, based on patient age, visual potential, and the presence of ocular comorbidities [17]. Various IOL implantation modalities have been developed for these cases, including anterior chamber IOLs, iris-claw or iris-sutured IOLs, sutured scleral-fixated IOLs (SSFIOLs), and posterior chamber IOLs [17].

SSFIOLs have been proposed as an effective means of correcting aphakia in pediatric patients lacking adequate capsular support [18]. In a study by Sen, P. et al. (2018), pediatric patients with congenital or traumatic lens subluxation underwent PPV with lens extraction followed by SSFIOL implantation using a four-point ab externo fixation technique [18]. The IOL implanted was a posterior chamber polymethyl methacrylate (PMMA) lens (Hanita) with a 6.5 mm optic diameter and a 13 mm overall diameter. Postoperative complications included choroidal detachment (2.86%), dispersed vitreous hemorrhage (2.86%), endophthalmitis (0.72%), elevated intraocular pressure (12.54%), diplopia (0.72%), retinal detachment (5.73%), and SSFIOL dislocation (4.6%) [18,19]. Despite these complications, best-corrected visual acuity (BCVA) was maintained or improved in 93.19% of eyes, supporting the effectiveness of this therapeutic approach [18].

Iris-sutures intraocular lenses are another viable surgical option to ensure adequate lens position [20]. Kopel et al. (2008) evaluated 22 eyes from 12 pediatric patients with ectopia lentis who underwent PPV and vitrectomy, with or without implantation of a foldable iris-sutured IOL [20]. All procedures were performed by a single vitreoretinal surgeon between 1998 and 2006. This study demonstrated that iris-fixated IOL implantation yielded visual outcomes comparable to those achieved with optically corrected aphakia, although the risk of IOL dislocation remained a significant concern [20].

In ectopia lentis, the choice of combined LV is primarily driven by the extent of lens instability, degree of capsular support, and age-appropriate IOL considerations [16]. Children with severe subluxation and inadequate zonular support benefit most from combined LV with scleral-fixated or iris-sutured IOLs [16,17,18,19,20].

### 4.3. Advanced Retinopathy of Prematurity (ROP)

ROP remains a leading cause of childhood blindness worldwide, especially in low-birthweight and preterm infants [21]. Early intervention with photocoagulation or intravitreal anti-VEGF therapy can prevent progression in many cases. However, advanced stages (Stage 4A, 4B, and 5 ROP) often require surgical intervention due to tractional retinal detachment (TRD) [21].

Surgical management in advanced cases typically involves vitrectomy or combined LV [22]. The need for lensectomy arises when fibrovascular proliferation extends anteriorly, obscuring the view or limiting access for membrane peeling and adequate traction release, or in cases with significant retrolental fibrosis [22]. When posterior structures can be safely visualized and accessed, LSV is preferred due to better visual and anatomic outcomes [22,23].

Sen et al. (2023) compared LSV and combined LV in a cohort of Stage 4 and 5 ROP eyes, demonstrating that LSV resulted in better visual outcomes and fewer postoperative complications [22]. Additionally, LSV has been associated with a lower incidence of glaucoma and amblyopia [22]. The combined LV group consisted of more complex cases, indicating that the surgical choice often reflects the underlying severity [22]. This is corroborated by data from Chang et al. (2024), who found that anatomic success was highest in Stage 4A (96.3%) and declined in more severe stages, with only 31.3% anatomic success in Stage 5 ROP [24]. Notably, the need for combined LV in Stage 4 eyes was significantly associated with poorer outcomes, suggesting that surgical complexity is a marker of worse prognosis [24].

In particularly severe cases, such as Stage 5C ROP (total retinal detachment along with anterior segment anomalies) with corneal opacification, a staged lensectomy and vitrectomy approach has been proposed [25]. Fei (2022) reported that performing lensectomy first, followed by delayed vitrectomy after corneal clearing, achieved partial retinal reattachment in 63.6% of eyes and restored corneal clarity in the majority, offering a strategic advantage when the posterior segment is initially inaccessible [25]. In this study, regular combined LV was not performed due to the invisible fundus. The average interval between the two procedures was 6.8 ± 4.6 months (2.5–18.5 months).

Modified surgical techniques have also evolved to minimize complications and improve access. Chandra et al. (2019) described a hybrid clear corneal micro-incision lensectomy and vitrectomy approach using 25G instruments in 50 eyes with Stage 5 ROP [26]. Sutureless closure without complications such as hypotony, flat anterior chamber, hyphema, or corneal edema was achieved [26]. This technique has been proposed as a safe and viable surgical alternative for Stage 5 ROP based on limited case series data, decreasing the high risk of iatrogenic breaks due to anterior retinal traction seen in traditional pars plana approaches [26].

Despite surgical advances, long-term complications such as glaucoma remain a concern [27,28]. Chandra (2019) and Nudleman (2017) both reported a higher risk of secondary glaucoma in patients undergoing combined LV, likely due to anterior segment disruption and increased inflammation [27,28]. Even LSV carries a risk, particularly in severe ROP stages [28].

Anatomic reattachment does not always correlate with functional vision [29,30]. Chehaibou et al. (2024) reported macular reattachment in 57.8% of eyes undergoing modified limbal LV, but only 64.1% achieved light perception or better [29]. Similarly, Rishi et al. (2019) presented a long-term follow-up of a patient with excellent anatomical outcome after combined LV who later developed Descemet’s membrane detachment 15 years postoperatively, illustrating that late sequelae can still compromise visual rehabilitation [30].

Collectively, the literature emphasizes that the surgical approach should be individualized based on disease stage, anterior segment clarity, and extent of fibrovascular proliferation [21,22,23,24,25,26,27,28,29,30]. While LSV remains the preferred option for less advanced disease, combined LV is indispensable in eyes with anterior TRD, retrolental fibrosis, or media opacities [22,23]. However, it must be approached cautiously given its association with poorer visual outcomes and higher postoperative morbidity.

### 4.4. Retinal Detachment (RD)

Rhegmatogenous retinal detachment (RRD) is a major vision-threatening condition [31]. RRD can result from retinal tears caused by trauma, structural retinal anomalies, pathological myopia, complicated cataract surgery, or posterior vitreous detachment [31]. Although RRD is more common in adults, retinal tears may also occur in younger patients, particularly after trauma or in association with hereditary collagen disorders [32].

Surgical repair of RRD using PPV has an anatomic success rate of approximately 80% [33]. A major complication of PPV in phakic eyes is the progressive development of cataracts, often necessitating cataract surgery, which may be technically more challenging in such cases [31].

LSV is preferred in pediatric patients, especially for cases involving TRD or opaque media [34]. Preserving the natural lens is critical in children to maintain accommodation. Therefore, LSV is often the procedure of choice when possible [31]. A retrospective analysis by Ferrone et al. (1997), which evaluated 85 eyes of 77 pediatric patients, found that 67% of lenses remained clear after LSV, while 15% developed cataracts and 18% required lens removal during subsequent surgery [34]. These findings suggest that LSV is effective in preserving lens clarity in the pediatric population [34].

Despite the advantages of LSV, complex retinal detachment cases in children may require combined LV [35,36]. Mendoza et al. (2025) reported a case of exudative RD in a 15-year-old female with Rubinstein–Taybi Syndrome (RTS) caused by a frameshift mutation in the cyclic AMP response element binding protein (CREBBP), who required combined LV [35]. In addition to classic RTS features (developmental delay, microcephaly, and broad thumbs and toes), the patient exhibited several ophthalmic manifestations, including left temporal retinal exudation, exudative RD, and inferotemporal hemorrhage. Despite initial improvement with multiple sessions of photocoagulation, focal TRD developed, necessitating combined LV. Similarly, Kawaguchi et al. (2025) reported two pediatric cases of RTS who developed TRD, both of whom underwent combined LV with IOL implantation [36]. In one case, retinal reattachment was achieved after three vitrectomies; in the other, reattachment could not be accomplished. Both patients developed poorly controlled glaucoma requiring surgical intervention. These cases highlight the complexity of RD management in RTS patients and suggest that treatment often requires multiple surgical interventions and carries an elevated risk of postoperative refractory glaucoma [35,36].

In pediatric RD, LSV is favored when visualization is adequate [34]. However, combined LV is required in cases with TRD or exudative RD where the lens obstructs access to the vitreous base or posterior pathology [34,35,36].

### 4.5. Persistent Fetal Vasculature (PFV)

PFV, previously called persistent hyperplastic primary vitreous (PHPV), is a rare but significant developmental anomaly arising from the failure of regression of the hyaloid vasculature, often leading to a spectrum of anterior and posterior ocular pathologies, including cataract, retrolental fibrovascular membranes, and RD [37]. Surgical intervention is frequently indicated in severe cases, particularly in combined PFV, where both anterior and posterior segments are affected [37]. In such situations, combined LV is often necessary to relieve the traction, clear the visual axis, and prevent further anatomic disruption [37].

Several studies have evaluated the safety and efficacy of combined LV for PFV, highlighting both the technical challenges and the potential for functional rehabilitation [38]. In a case series by Lyu et al. (2020) involving 19 eyes with unilateral combined PFV, patients underwent limbal lensectomy, capsulotomy, anterior vitrectomy, dissection of the retrolental membrane and stalk, and in-the-bag IOL implantation [38]. In this study, 95% of IOLs remained well positioned, and retinal dragging was reversed in all 8 eyes with preoperative peripapillary traction. While 47% of eyes achieved BCVA better than 20/200, poorer outcomes were associated with baseline peripapillary retinopathy, emphasizing the prognostic importance of initial retinal health [38]. These findings underscore the feasibility of combined LV with IOL implantation as an alternative to treat eyes with combined PFV, with long-term refractive monitoring required due to postoperative myopic shift [38].

Similarly, Khurana et al. (2021) reported favorable outcomes in a prospective cohort of 20 children undergoing phacoaspiration with or without IOL implantation, combined with dissection and cauterization of the PFV stalk [39]. Good visual fixation (central, steady, maintained) was achieved in 80% of patients, and no cases of intraoperative bleeding, glaucoma, or retinal detachment occurred [39]. The major complication was VAO, requiring membranectomy in 8 children. Visual outcomes were more guarded in children with microphthalmia, aphakia, or combined PFV, reinforcing the importance of early surgical timing and aggressive amblyopia therapy [39].

Even though anatomic restoration is vital, it does not always lead to visual improvement [37]. Loukovaara et al. (2024) found that despite combined LV surgery to address both anterior and posterior PFV components in pediatric patients with unilateral congenital cataract and PFV, visual outcomes were modest [37]. The best result was a visual acuity of 0.5 (20/40 on the Snellen chart) in one child, while others ranged from finger counting to light perception. Contributing factors included amblyopia, microphthalmia, and macular involvement. Postoperative complications such as secondary cataracts and esotropia were also reported, underscoring the need for careful preoperative evaluation and long-term visual rehabilitation strategies [37].

Age and lens status are pivotal in surgical decision-making [40]. In Huang, H.C. et al. (2023), patients with posterior or combined PFV underwent either LSV or combined LV depending on the extent of anterior segment involvement, such as the presence of cataract or lens opacification that obstructed the visual axis or hindered safe access to the PFV stalk [40]. IOL implantation was avoided in children under 2 years due to higher complication risk and concern for ocular growth interference [40]. Only 26.3% of eyes achieved vision better than counting fingers, while 29% suffered poor outcomes including no light perception. However, a significant benefit of surgery was greater axial elongation in operated eyes, suggesting improved cosmetic outcomes through enhanced ocular growth even when visual rehabilitation was limited [40].

Surgical approach also influences outcomes. In a 20-year retrospective study, Bata et al. (2019) found that a limbal approach resulted in better visual acuity and lower complication rates compared to the pars plana approach [41]. Among 58 infants undergoing early combined LV before 7 months of age, 43% of eyes treated via limbal access achieved BCVA better than 1.0 logMAR, compared to only 11% via pars plana access [41]. Importantly, RD occurred significantly more in the latter group, highlighting the potential safety advantages of anterior access in specific cases [41].

Innovative surgical tools, such as endoscopic-assisted vitrectomy, are expanding the therapeutic armamentarium [42]. Otsubo et al. (2024) reported the utility of 23-gauge rigid endoscopes in enhancing posterior segment visualization in eyes with media opacity, enabling safer and more effective PFV stalk dissection [42]. No significant complications occurred in their series, suggesting endoscopic techniques may be particularly valuable in challenging PFV presentations [42].

Although visual prognosis in PFV is variable and often guarded, especially in eyes with posterior involvement or structural anomalies, surgical intervention can still yield meaningful functional and anatomical benefits in select cases [43]. As Soheilian M., et al. (2002) emphasized in their review of 54 eyes, visual improvement was possible in patients with anterior or combined PFV and a relatively preserved retina [43]. Surgical therapy should thus be individualized. The extent of fibrovascular proliferation, ocular morphology, patient age, and potential for amblyopia should guide decision-making [43].

Combined LV is frequently used in the surgical management of PFV, especially in cases involving both anterior and posterior segments, or when significant lens opacity or retrolental traction is present [37,38,39,40,41,42,43]. However, high-level comparative data are limited. While long-term visual outcomes vary, early and appropriately tailored intervention offers the best opportunity for anatomical success, cosmetic improvement, and functional vision [40].

A comparative overview of the clinical indications, anatomical and visual outcomes, and complications of combined LV across common pediatric pathologies is provided in Table 1.

Given the variety of IOL implantation techniques available for pediatric patients undergoing combined LV surgery, Table 2 summarizes these strategies, including their surgical contexts, associated outcomes, and common complications.

Limitations of this review include the low frequency and clinical complexity of the ocular conditions discussed, which limit the availability of robust data on this topic. Most of the included studies are retrospective and show variability in patient populations, surgical techniques, outcome measures, and follow-up durations. Additionally, many are single-center studies with small sample sizes, which may affect the generalizability of findings. Despite these limitations, this review provides a structured summary of the current literature on combined LV in pediatric patients, organized by clinical indication. It offers practical insight into when and why combined LV may be appropriate in children and highlights areas where future research is needed.

## 5. Conclusions

This review underscores the importance of a tailored, case-by-case surgical approach when managing pediatric patients requiring cataract extraction, lensectomy, and/or vitrectomy. While LSV is often preferred to preserve accommodation, combined LV procedures are frequently necessary in the presence of complex anterior-posterior segment pathology.

Outcomes following combined LV in children remain highly variable and are influenced by multiple factors, including patient age, underlying ocular anatomy, disease severity, and surgical technique. Although anatomic success is commonly achievable, functional visual outcomes are often limited. Moreover, combined LV is associated with a higher risk of postoperative complications such as glaucoma, VAO, and IOL dislocation, underscoring the need for long-term follow-up and comprehensive visual rehabilitation strategies.

Future studies and harmonized multicenter registries are essential to refine surgical indications and improve outcomes in this complex pediatric population. Although randomized clinical trials provide the highest level of evidence, their design and implementation in this context are challenging due to the rarity of combined LV procedures in children, the heterogeneity of underlying conditions, and the need for long-term follow-up to assess visual outcomes. However, we believe that outcome variability can be partially addressed through collaborative, prospective data collection. Rather than advocating for a universal protocol, future efforts should focus on establishing clinical principles based on shared outcome predictors such as patient age, ocular anatomy, and surgical indication. We propose the implementation of retrospective analyses using large databases such as the IRIS Registry or the Vestrum Health database. These resources could enable meaningful evaluations of surgical outcomes and complications in pediatric patients, supporting more consistent and informed decision-making while preserving individualized patient care.

## Figures and Tables

**Table 1:** Summary of indications and outcomes for combined lensectomy and vitrectomy (LV) in pediatric pathologies.

Pediatric Condition	Indication for Combined LV	Reported Anatomic Outcome	Reported Visual Outcome	Common Complications	Key References
Congenital cataracts	1. Bilateral dense cataracts with posterior segment involvement. 2. VAO prevention. 3. Better posterior access	High rate of IOL stability; successful visual axis clearing in most cases	Mean BCVA 0.24 logMAR; poorer in children < 2 years; 14.9% with low vision	VAO, IOL pupillary capture, iris incarceration, and glaucoma	[10,13]
Ectopia lentis	1. Lens subluxation due to trauma or systemic conditions 2. Inadequate capsular support	Variable based on IOL type and fixation technique Higher rate of IOL dislocation in iris-fixation vs. scleral fixation	BCVA maintained or improved in 93.19% of eyes. Good visual outcome if no amblyopia or retinal pathology	IOL dislocation, elevated IOP, RD, endophthalmitis	[18,19,20]
ROP	Advanced stages (4/5) with anterior fibrovascular proliferation, retrolental fibrosis, or poor media clarity	96.3–31.3% retinal reattachment depending on stage. Better in Stage 4A than 5	Light perception or better in 64.1%; BCVA correlated poorly with anatomical outcome	Glaucoma, VAO, intraoperative/postoperative hemorrhage	[22,24,25]
RD	Complex RDs with TRD, exudative changes, or opaque media requiring lens removal for adequate access	Variable. In some cases, anatomical reattachment required multiple surgeries	Generally poor outcomes	Glaucoma, retinal re-detachment	[35,36]
PFV	1. Significant anterior/posterior pathology 2. Cataract with retrolental membranes and stalk traction	Retinal traction relief in majority; greater axial elongation	Variable, depending on pathology extent. ~47% achieved BCVA better than 20/200; best outcomes when macula spared, and early surgery performed	Postoperative myopic shift, VAO, RD	[38,39,41]

**Table 2:** Comparison of intraocular lens (IOL) implantation strategies in pediatric combined surgery.

IOL Technique	Surgical Indication	Patient Age Group	Visual Outcomes	Complications	Advantages	Key References
In-the-bag IOL	Congenital cataracts with adequate capsular support	Typically > 2 years	Mean BCVA ~0.24 logMAR; 85% achieved stable VA	VAO, pupillary capture, glaucoma, refractive shift	Preferred when capsular support intact; facilitates central fixation	[10,12]
Scleral-fixated IOL	Inadequate capsular support	Often > 5 years	93% maintained or improved BCVA postoperatively	IOL dislocation, elevated IOP, RD, endophthalmitis	Secure fixation without iris manipulation; avoids anterior chamber crowding	[18]
Iris-sutured IOL	Inadequate capsular support	Often > 5 years	Comparable to aphakic correction; acceptable BCVA in most patients	IOL decentration, pigment dispersion, glaucoma risk, chronic uveitis	Avoids scleral suturing; suitable when scleral fixation not possible	[20]
Aphakia (no IOL implanted)	Infants < 2 years old, or eyes with microphthalmia, severe PFV, or poor prognosis	<2 years or cases with ocular growth concerns	Limited in many cases, depending on comorbidities; amblyopia risk	VAO; glaucoma; need for secondary IOL later; rehabilitation burden	Avoids IOL complications; allows ocular growth	[39,40]
